# Association of Underweight, Sarcopenia, and Cancer Cachexia with Survival Outcomes in Hypopharyngeal Cancer Radiotherapy

**DOI:** 10.3390/cancers18081244

**Published:** 2026-04-14

**Authors:** Natsuo Tomita, Daisuke Kawakita, Takuma Matoba, Kiyoshi Minohara, Sho Iwaki, Koji Tsukamoto, Masanosuke Oguri, Nozomi Kita, Akira Torii, Masanari Niwa, Dai Okazaki, Taiki Takaoka, Shinichi Iwasaki, Akio Hiwatashi

**Affiliations:** 1 Department of Radiology, Nagoya City University Graduate School of Medical Sciences, 1 Kawasumi, Mizuho-cho, Mizuho-ku, Nagoya 467-8601, Aichi, Japan; 2 Department of Otolaryngology, Head and Neck Surgery, Nagoya City University Graduate School of Medical Sciences, 1 Kawasumi, Mizuho-cho, Mizuho-ku, Nagoya 467-8601, Aichi, Japan

**Keywords:** underweight, sarcopenia, cancer cachexia, hypopharyngeal cancer, head and neck cancer, radiotherapy

## Abstract

**Objectives:** This study investigates the association of pretreatment underweight, sarcopenia, and cancer cachexia with survival outcome in hypopharyngeal cancer (HPC) radiotherapy. **Methods:** This retrospective observational study analyzed 167 patients with newly diagnosed HPC treated with definitive radiotherapy. The definitions of underweight, sarcopenia, and cancer cachexia are based on the international consensus of the European Palliative Care Research Collaborative. Underweight and sarcopenia were analyzed in all 167 patients, while cachexia analyses were restricted to the 117 patients for whom pretreatment weight-loss data were available. Survival outcomes were estimated using the Kaplan–Meier method and compared using the log-rank test, and subsequently analyzed using multivariate Cox proportional hazards models. **Results:** The median follow-up period was 28 months. Cachexia analyses were restricted to the 117 patients for whom pretreatment weight-loss data were available; of these, 45 (38%) met criteria for cancer cachexia. Patients with underweight (*n* = 76, 46%) or cancer cachexia had significantly lower locoregional control, disease-free survival, and overall survival compared to those not underweight and without cachexia, respectively, whereas there was no difference in any outcome between patients with sarcopenia (*n* = 54, 32%) and those without. Given the definitional overlap among underweight, sarcopenia, and cachexia, these three variables were entered into the multivariate analysis separately—which included age, sex, performance status, double cancer, T-classification, N-classification, chemotherapy administration, treatment era, and radiation dose—confirming that underweight and cancer cachexia remained independently associated with worse LRC, DFS, and OS. In the fully adjusted multivariate Cox proportional hazards models, the hazard ratios for mortality risk were 1.9 (95% confidence interval [CI], 1.1–3.4; *p* = 0.030) and 2.0 (95% CI, 1.1–3.8; *p* = 0.032) for patients with underweight or cancer cachexia, respectively. **Conclusions:** Pretreatment underweight and cancer cachexia negatively impact survival outcomes, including locoregional control, in HPC radiotherapy. Prospective studies with standardized nutritional assessment protocols, pre-specified intervention arms, and sufficient sample sizes are essential to validate these findings and to establish the clinical benefit of pre-treatment nutritional optimization in this patient population.

## Simple Summary

We examined the association of pretreatment underweight, sarcopenia, and cancer cachexia with survival outcomes in 167 patients with hypopharyngeal cancer undergoing radiation therapy. The prevalence of pretreatment underweight, sarcopenia, and cachexia among the hypopharyngeal cancer patients included in this analysis was 46%, 32%, and 38%, respectively. Our results show that pretreatment underweight and cancer cachexia are independent negative prognostic factors for locoregional control, and for disease-free survival and overall survival. Prospective studies with standardized nutritional assessment protocol are warranted to establish the clinical benefit of pre-treatment nutritional optimization in this patient population.

## 1. Introduction

Body mass index (BMI) is recognized as a risk indicator for the development of many types of cancer and for cancer treatment itself [1,2]. Large-scale epidemiological studies and meta-analyses indicate that a low BMI (i.e., underweight) is generally associated with lower disease-free survival (DFS) and overall survival (OS) across many cancer types [2,3]. Underweight is associated with malnutrition and reduced muscle mass, such as sarcopenia. These conditions can lead to a poor prognosis due to reduced treatment tolerance and an increased risk of adverse events (AEs) [4,5]. Furthermore, underweight corresponds with cancer cachexia [6] and has a profound impact on survival outcomes [7,8,9,10]. The European Palliative Care Research Collaborative (EPCRC) published a definition and diagnostic criteria for cancer cachexia in 2011. This consensus established underweight and sarcopenia as one of the diagnostic criteria for cancer cachexia alongside a weight loss of at least 2% [11].

Between 42% and 77% of patients with advanced head and neck cancer (HNC) are malnourished due to difficulties with chewing and swallowing, which are either a direct result of the disease itself or a symptom of cancer cachexia [12,13]. In radiation therapy (RT), where function preservation is one of the primary goals, locoregional control (LRC) is essential for quality of life [14]. Therefore, it is crucial to investigate whether underweight or cancer cachexia in HNC patients affects survival outcomes, including LRC, and the extent and causes of this impact when considering HNC treatment strategies. Hypopharyngeal cancer (HPC) is not only the most lethal HNC subsite but is also associated with particularly high rates of dysphagia and malnutrition due to hypopharyngeal dysfunction, making it a clinically important model for investigating nutritional prognostic factors [15]. Underweight and cancer cachexia may impair locoregional tumor control through multiple biological mechanisms. Malnutrition and cachexia have been associated with impaired anti-tumor immune surveillance [16], reduced DNA repair capacity [17], and metabolic alterations of the tumor microenvironment [18], all of which may reduce the effectiveness of radiotherapy-mediated locoregional tumor control. Cachexia-associated immune dysfunction and systemic inflammation may further compromise the host’s ability to achieve and sustain locoregional tumor control independent of distant disease.

We hypothesized that (1) pretreatment underweight, sarcopenia, and cancer cachexia would each be independently associated with worse LRC, DFS, and OS after multivariate adjustment for established prognostic factors, and (2) that the observed prognostic differences between groups would not be fully attributable to differences in treatment tolerance or AEs. The primary objective of this study is to clarify the association between underweight, sarcopenia, and cancer cachexia and the survival outcomes of LRC, DFS, and OS in HPC patients. A secondary objective is to investigate the correlation between the three aforementioned factors and treatment tolerance and AEs. Prior studies have typically evaluated the prognostic impact of BMI or underweight [2,3], sarcopenia [4,5], or cancer cachexia [7,8,9,10] as isolated factors. However, because these conditions are interrelated by definition—cachexia criteria incorporate both underweight and sarcopenia—analyzing each factor independently and simultaneously is essential to disentangle their individual contributions. To date, no prior study has simultaneously and individually analyzed whether underweight, sarcopenia, and cancer cachexia are each independently associated with LRC, DFS, and OS in HPC patients treated with RT.

## 2. Materials and Methods

### 2.1. Study Population

#### 2.1.1. Patients

The selection criteria for this retrospective observational study were as follows: (1) Patients with a histologically confirmed diagnosis of primary HPC between January 2004 and March 2025. (2) Newly diagnosed HPC patients without distant metastases. (3) Patients scheduled for definitive RT, even if they discontinued RT due to AEs. The exclusion criteria were as follows: (1) Patients who received postoperative or palliative RT. (2) Patients who received RT for locoregional recurrence after surgery. (3) Patients with double cancer other than HPC, for whom curative treatment for double cancer was not planned. This study included 167 patients who met the criteria. Table 1 shows a summary of patient and treatment characteristics. The staging classification was standardized based on the eighth edition of the TNM classification. This study was approved by the Nagoya City University Graduate School of Medicine Institutional Review Board (approval number 60-25-0166). Informed consent was obtained via an opt-out website due to the retrospective observational nature of the analysis.

#### 2.1.2. Definition of Underweight, Sarcopenia, and Cancer Cachexia

According to the standard [11], the BMI cutoff value for underweight was set at 20.0 kg/m^2^. Sarcopenia was diagnosed by measuring the skeletal muscle area at the L3 vertebra level using a CT scan with SYNAPSE VINCENT software version 7.2 (Fujifilm, Tokyo, Japan), and dividing this value by the patient’s height squared to calculate the skeletal muscle index (SMI). The SMI cutoff values for sarcopenia were 39.8 cm^2^/m^2^ for men and 28.4 cm^2^/m^2^ for women, which is consistent with values established for East Asians [19]. These East Asian-specific cutoff values are appropriate for the Japanese population studied herein. According to the EPCRC criteria [11], the definition of cancer cachexia is met when any one of the following three conditions is satisfied: (1) Weight loss surpassing 5% over the past 6 months. (2) BMI < 20.0 kg/m^2^ and weight loss > 2%. (3) Sarcopenia and weight loss > 2%.

### 2.2. Treatment Characteristics

#### 2.2.1. Radiotherapy

A previous paper describes the method of CT imaging for RT planning and configuring the planning target volume (PTV) in detail [20,21]. An adaptive, two-step RT method was used, and elective nodal regions were excluded from the cutdown field plan. Since 2012, treatment has primarily used intensity-modulated radiation therapy (IMRT), whereas prior to 2012, treatment used three-dimensional conformal radiation therapy (3DCRT). Of the total number of patients, 118 (71%) were treated with IMRT, while 49 (29%) were treated with 3DCRT. IMRT used a 6 MV X-ray beam delivered by Helical Tomotherapy (Accuray, Sunnyvale, CA, USA) or Radixact (Accuray, Sunnyvale, CA, USA), while 3DCRT used Clinac-ix or TrueBeam (Varian, Palo Alto, CA, USA). IMRT was planned so that 50% of the PTV would receive the full prescribed dose. 3DCRT was performed using an isocenter prescription. The median fractional dose was 2.0 Gy (range 1.8–3.0 Gy), and the doses were described as equivalent 2 Gy fractions (EQD2) using the linear quadratic model with an α/β value of 10 Gy. RT was administered to the PTV with a median dose of 70 Gy (range 30.0–70.0 Gy). Only one patient completed treatment at 30 Gy; this patient discontinued treatment due to bacterial pneumonia during RT. All other patients received treatment at a dose equivalent to at least 60 Gy. The cutout field plan change was implemented at a median dose of 46 Gy (range 30–49.7 Gy).

#### 2.2.2. Chemotherapy

Eighty percent of patients (*n* = 133) received chemotherapy. Twenty percent of the patients (*n* = 34) did not receive chemotherapy for reasons that included early-stage cancer, low renal function, advanced age, and patient refusal. Chemotherapy was most commonly administered concurrently with RT (*n* = 72, 54%), followed by induction before RT and concurrently with RT (*n* = 48, 36%), and then induction before RT only (*n* = 13, 10%). Of the patients who received chemotherapy concurrently with RT, half (*n* = 65, 54%) received cisplatin (80–100 mg/m^2^ every 3 weeks during RT, 2–3 cycles), 29% (*n* = 29) received cetuximab (400 mg/m^2^ one week before the start of RT, followed by 250 mg/m^2^ every week during the RT period, 8 cycles), and 22% (*n* = 26) received docetaxel (10 mg/m^2^ every week during RT, 6 cycles). Among the patients who received induction chemotherapy before RT, 44 (72%) received a docetaxel (75 mg/m^2^, day 1), cisplatin (75 mg/m^2^, day 1), and fluorouracil regimen (750 mg/m^2^, day 1–5) (TPF every 3 weeks, 1–3 cycles), and 17 (28%) received a cisplatin (100 mg/m^2^, day 1) and fluorouracil regimen (1000 mg/m^2^, day 1–4) (PF every 3 weeks, 1–3 cycles). Given the heterogeneity of chemotherapy regimens, chemotherapy administration was included as a binary covariate in multivariate models, which represents a simplification. While the inclusion of chemotherapy as a binary covariate captures whether systemic therapy was administered, it does not account for differences in regimen type (cisplatin, cetuximab, docetaxel, or induction protocols), dosing intensity, or number of cycles. This simplification may obscure differential treatment effects across subgroups, particularly as the cachexia group had a significantly lower rate of chemotherapy use (31% vs. 10%, *p* = 0.006). This represents a recognized limitation of the present analysis.

### 2.3. Statistical Analysis

The follow-up duration was determined from the start of RT. For patients who received induction chemotherapy, the follow-up duration was determined from the start of that treatment. LRC was defined as the time from the start of treatment until locoregional recurrence. DFS was defined as the time from treatment initiation until overall recurrence or death. OS was calculated from the start of treatment until the last follow-up or death. The Kaplan–Meier method was used to estimate survival, and the log-rank test was used to compare survival estimates between groups. Then, a Cox proportional hazards model was used for a multivariate analysis to evaluate prognostic factors for LRC, DFS, and OS. Given the known conceptual and definitional overlap between underweight, sarcopenia, and cachexia, these three variables were entered into separate multivariate models rather than simultaneously, thereby precluding the need for formal multicollinearity diagnostics between these specific variables. Among the remaining covariates entered jointly, variance inflation factors were assessed and no evidence of clinically meaningful multicollinearity was detected (all variance inflation factors < 5). The 10 covariates used in the adjustment were: age, sex, performance status (PS), double cancer, smoking history, T-classification, N-classification, chemotherapy administration, treatment era, and RT dose. These covariates are major prognostic factors in RT for HPC [14,20]. Treatment era (before vs. after 2012) was included a priori as a covariate to account for the temporal shift from 3DCRT to IMRT and associated advances in treatment planning, imaging, and supportive care over the study period. No statistically significant violations of the proportional hazards assumption were identified (all *p* > 0.05 for Schoenfeld residual tests). Given the known conceptual and definitional overlap between underweight, sarcopenia, and cachexia, these three variables were entered into separate multivariate models rather than simultaneously, thereby precluding the need for formal multicollinearity diagnostics between these specific variables. Among the remaining covariates entered jointly, variance inflation factors were assessed and no evidence of clinically meaningful multicollinearity was detected (all variance inflation factors < 5). AEs were evaluated using the Common Terminology Criteria for Adverse Events (CTCAE) version 5.0. The Gray test was used to compare the occurrence of late AEs between groups. Various statistical tests were used to compare factors among groups, including chi-square, Fisher’s exact, and *t*-tests. As pretreatment weight-loss data were missing for 50 patients (30%), these patients were excluded from the cachexia-specific analyses. Underweight and sarcopenia were analyzed in all 167 patients. For covariates with missing values, we used the multiple imputation method to create 50 imputed datasets. We applied the Cox proportional hazards model to each dataset and then combined the results using Rubin’s rules. Furthermore, as a sensitivity analysis, we performed multivariate analyses for two scenarios: one assuming that all missing values were for cachexia and the other assuming they were for non-cachexia. All statistical analyses were performed using EZR (version 3.6.3; R Foundation for Statistical Computing, Vienna, Austria), a graphical user interface for R [22]. If there are missing values in any of the required variables, those data points are automatically excluded from the analysis (i.e., listwise deletion). The significance threshold was set at *p* < 0.05.

## 3. Results

### 3.1. Patient Characteristics

**Figure 1:**
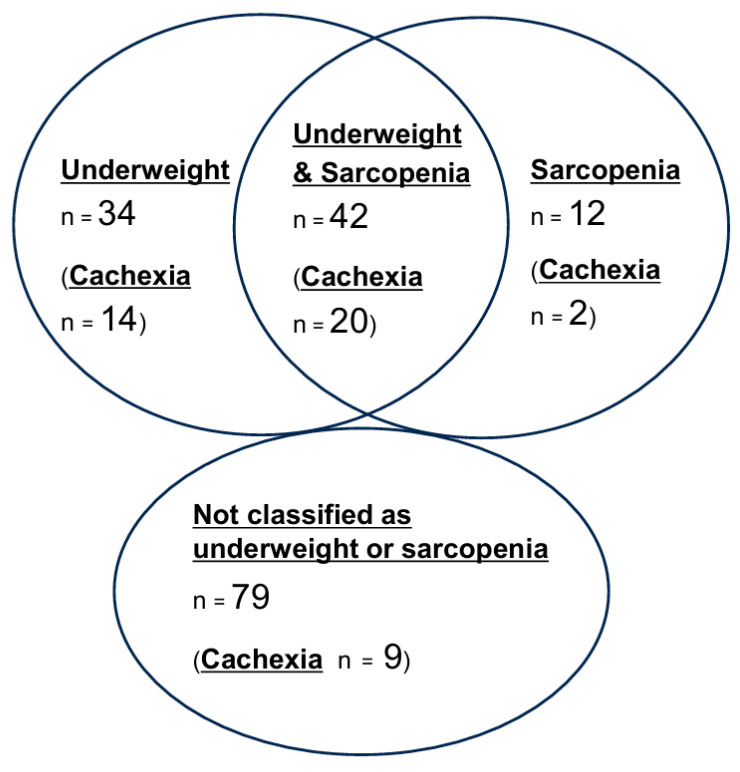
Breakdown of patients categorized as underweight, sarcopenia, or cancer cachexia. The presence of underweight and sarcopenia was evaluated in all 167 patients, while assessment of the presence of cachexia was restricted to the 117 patients for whom complete weight-loss data were available.

All patients had squamous-cell carcinoma (SCC). Table 1 shows the characteristics of the patients and their treatments. The median age was 69 years. Most patients (92%) had a favorable PS of 0 or 1. Double cancer other than HPC was identified in 27 patients (16%), with 19 of these cases being esophageal cancer. Additionally, six patients had gastric cancer, three had HNC other than HPC, two had liver cancer, and others had bladder or breast cancer. Three patients had two concurrent cancers, and one patient had three. Most patients underwent curative treatment with surgery or chemoradiation for their double cancer, while two patients with early-stage double cancers were observed because treatment for the primary cancer was prioritized. The presence of underweight and sarcopenia was evaluated in all 167 patients, while the assessment of the presence of cachexia was restricted to the 117 patients for whom complete weight-loss data were available. There were 76 patients (46%) who were underweight and 54 patients (32%) with sarcopenia. Forty-five patients (38%) were determined to be in a state of cancer cachexia. Figure 1 shows the breakdown of patients with underweight, sarcopenia, and cancer cachexia.

A comparison of patient characteristics revealed that the underweight group had a higher proportion of females, sarcopenia, and cancer cachexia than the non-underweight group (all *p* < 0.001). Treatment without chemotherapy or with doses lower than 66 Gy was more common in the underweight group than the non-underweight group (*p* = 0.087, 0.066, respectively). The cancer cachexia group was older and had a higher proportion of PS 1–2, sarcopenia, and the non-use of chemotherapy than the non-cachexia group (*p* = 0.048, 0.012, 0.010, and 0.006, respectively), as shown in Table 1. In the multivariate analysis, we adjusted for 10 covariates of age, sex, PS, double cancer, smoking history, T-classification, N-classification, chemotherapy administration, treatment era, and radiation dose to account for these imbalances across groups. Supplementary Table S1 shows the comparison of patient and treatment characteristics between patients with available pretreatment weight-loss data (evaluable for cachexia, *n* = 117) and patients with missing weight-loss data (*n* = 50). The two groups were broadly comparable in terms of age, sex, PS, double cancer, smoking history, T-classification, chemotherapy administration, treatment era, and radiation dose. Only N-classification was different between the two groups (*p* = 0.005).

### 3.2. Outcomes

The median follow-up period was 28 months (range 1–199) for all patients and 36 months (range 1–199) for those alive. Of the total cohort, 60 patients (36%) died, including 43 (26%) from HPC and 17 (10%) from other causes. A total of 47 patients (28%) experienced local recurrence, 32 patients (19%) experienced regional recurrence, and 20 patients (12%) experienced distant metastases. The 3-year rates of LRC, DFS, and OS were 59% (95% confidence interval [CI], 50–67), 49% (95% CI, 40–57), and 70% (95% CI, 62–78), respectively.

### 3.3. Univariate and Multivariate Analyses for LRC, DFS, and OS

**Figure 2:**
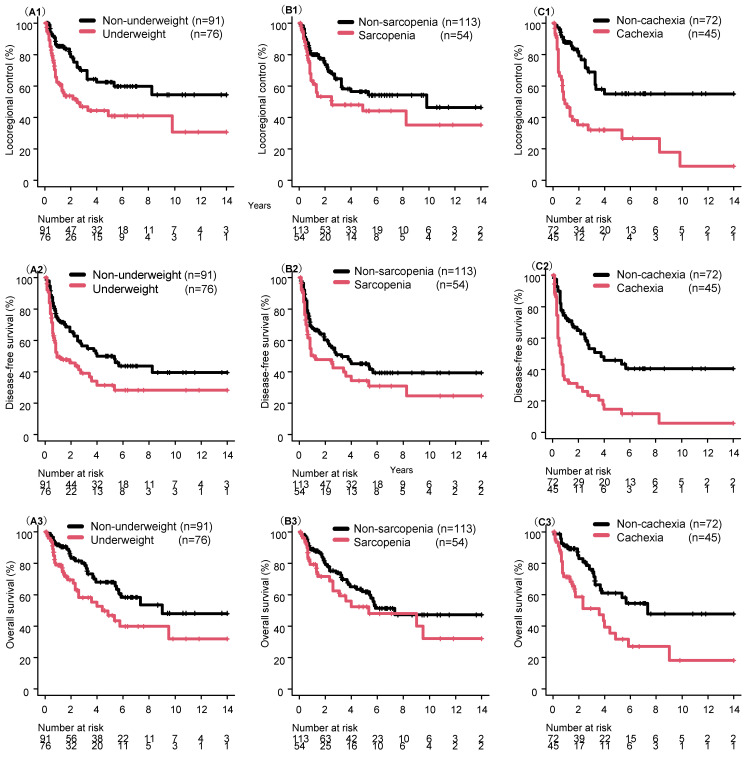
Comparison of survival curves for locoregional control (LRC), disease-free survival (DFS), and overall survival (OS) between the underweight and non-underweight (**A1**–**A3**), sarcopenia and non-sarcopenia (**B1**–**B3**), and cancer cachexia and non-cachexia (**C1**–**C3**) groups. Underweight and sarcopenia were analyzed in all 167 patients, while cachexia analyses were restricted to the 117 patients for whom complete weight-loss data were available.

Figure 2 shows survival curve comparisons between the underweight and non-underweight (2A1–2A3), sarcopenia and non-sarcopenia (2B1–2B3), and cancer cachexia and non-cachexia (2C1–2C3) groups, respectively. LRC, DFS, and OS were lower in the underweight group than in the non-underweight group (*p* = 0.002, 0.008, and 0.014, respectively). LRC, DFS, and OS did not differ statistically between the sarcopenia and non-sarcopenia groups (*p* = 0.069, 0.076, and 0.18, respectively), although the observed trends may warrant attention in future, adequately powered studies. LRC, DFS, and OS were lower in the cancer cachexia group than in the non-cachexia group (*p* < 0.001, *p* = 0.001, and *p* = 0.003, respectively). Table 2 shows the results of the univariate analyses for LRC, DFS, and OS. PS, N-classification, and chemotherapy administration showed significant results in at least two of the three outcomes.

**Figure 3:**
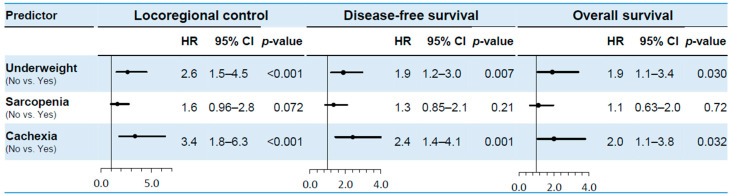
Results of multivariate analyses for locoregional control (LRC), disease-free survival (DFS), and overall survival (OS). Underweight and sarcopenia were analyzed in all 167 patients, while cachexia analyses were restricted to the 117 patients for whom complete weight-loss data were available. HR, hazard ratio; CI, confidence interval.

Underweight and sarcopenia were analyzed in all 167 patients, while cachexia analyses were restricted to the 117 patients for whom complete weight-loss data were available. Given the known conceptual and definitional overlap between underweight, sarcopenia, and cachexia (as illustrated in Figure 1), these three variables were entered into the model separately, adjusting for 10 covariates of age, sex, PS, double cancer, smoking history, T-classification, N-classification, chemotherapy administration, treatment era, and RT dose. Supplementary Table S2 presents hazard ratios (HRs), 95% CIs, and *p*-values for all covariates included in each multivariate Cox proportional hazards analysis. Figure 3 shows the results of the multivariate analyses for LRC, DFS, and OS. Underweight status remained independently associated with worse LRC, DFS, and OS outcomes (HR 2.6, 95% CI 1.5–4.5, *p* < 0.001; HR 1.9, 95% CI 1.2–3.0, *p* = 0.007; and HR 1.9, 95% CI 1.1–3.4, *p* = 0.030, respectively). There was no statistically significant evidence of an independent association between sarcopenia and any of the three outcomes in the multivariate analysis (HR 1.6, 95% CI 0.96–2.8, *p* = 0.072; HR 1.3, 95% CI 0.85–2.1, *p* = 0.21; and HR 1.1, 95% CI 0.63–2.0, *p* = 0.72, respectively). Cancer cachexia remained independently associated with worse LRC, DFS, and OS (HR 3.4, 95% CI 1.8–6.3, *p* < 0.001; HR 2.4, 95% CI 1.4–4.1, *p* = 0.001; and HR 2.0, 95% CI 1.1–3.8, *p* = 0.032, respectively). Supplementary Tables S3 and S4 show the results of multiple imputation and a sensitivity analysis. In all analyses—including the complete-case analysis, multiple imputation, and a sensitivity analysis —the direction of the hazard ratio for the primary exposure was consistent, and the results were generally robust.

### 3.4. Treatment Tolerance

Compared to the non-underweight group, there was a tendency toward the administration of chemotherapy being less common (26% vs. 15%, *p* = 0.087) and a lower radiation dose, below 66 Gy (14% vs. 7%, *p* = 0.066), in the underweight group. There was no statistically significant evidence in the median overall RT periods between the underweight and non-underweight groups (50 days [range 26–86] vs. 50 days [range 20–71], *p* = 0.15). The cancer cachexia group had a lower rate for the administration of chemotherapy than the non-cachexia group (31% vs. 10%, *p* = 0.006). There was no statistically significant evidence in the use of a lower radiation dose, below 66 Gy, between the cancer cachexia and non-cachexia groups (13% vs. 10%, *p* = 0.56). There was no statistically significant evidence in the median RT periods between the cancer cachexia and non-cachexia groups (50 days [range 26–86] vs. 50 days [range 20–71], *p* = 0.69).

### 3.5. Late Adverse Events

Thirty-one patients (19%) experienced grade 2 or higher late AEs, and four of those patients experienced separate grade 2 and grade 3 AEs. Twenty-three (14%) of those patients experienced grade 2 AEs, 11 (6.6%) experienced grade 3 AEs, 0 experienced grade 4 AEs, and one (0.6%) experienced grade 5 AEs. Grade 2 AEs included hypothyroidism (*n* = 17), stroke (*n* = 2), dysphagia (*n* = 1), dry mouth (*n* = 1), neck pain (*n* = 1), recurrent laryngeal nerve palsy (*n* = 1), and hypoparathyroidism (*n* = 1). Grade 3 AEs included stroke (*n* = 3), aspiration (*n* = 3), pneumonitis (*n* = 2), dysphagia (*n* = 1), recurrent laryngeal nerve palsy (*n* = 1), and osteonecrosis of the jaw (*n* = 1). A grade 5 AE was an unexplained death that occurred one week after completing cetuximab-combined RT in a T4N2cM0 patient who was neither underweight nor cachectic.

There was no statistically significant evidence in the three-year rates of ≥grade 2 and ≥grade 3 late AEs between the underweight and non-underweight groups (≥grade 2, 16% vs. 23%, *p* = 0.11; ≥grade 3, 6.2% vs. 7.9%, *p* = 0.40). There was no statistically significant evidence in the three-year rates of grade 2 and 3 late AEs between the cancer cachexia and non-cachexia groups (≥grade 2, 29% vs. 18%, *p* = 0.76; ≥grade 3, 8.3% vs. 1%, *p* = 0.46).

## 4. Discussion

To the best of our knowledge, this is among the first studies to simultaneously and separately analyze the independent associations of underweight, sarcopenia, and cancer cachexia with LRC, DFS, and OS in HPC patients undergoing RT. As shown in Figure 1, these three factors are closely interrelated in cancer patients; therefore, it is not possible to determine which factor specifically influences prognosis without deliberately analyzing them separately. Our study shows that underweight and cancer cachexia were the independent risk factors for all outcomes of LRC, DFS, and OS in this patient population. The underweight and non-underweight groups were balanced in terms of most factors, except for gender, with females typically experiencing potential benefits in terms of survival outcomes. Nevertheless, the underweight group showed inferior outcomes compared to the non-underweight group. The HRs for locoregional recurrence and mortality risk were 2.6 (95% CI, 1.5–4.5; *p* < 0.001) and 1.9 (95% CI, 1.1–3.4; *p* = 0.030) for patients with underweight. The observed association between underweight and worse LRC is clinically important but mechanistically complex. Potential mechanisms may include impaired anti-tumor immune surveillance [16], reduced DNA repair capacity [17], and alterations to the tumor microenvironment associated with metabolic derangement [18]. However, LRC may be influenced by multiple factors, including treatment intensity, compliance, and comorbidities, and our observational data cannot disentangle these contributions.

The association between underweight and poor prognosis is commonly attributed to cancer cachexia, a condition characterized by weight loss and muscle wasting. In fact, the proportions of patients with cancer cachexia were 45% in the underweight group vs. 12% in the non-underweight group (*p* < 0.001). Although differences were observed in several patient background characteristics between the cancer cachexia and non-cachexia groups, multivariate analysis revealed that the cancer cachexia group also significantly impacted treatment outcomes. The HR for mortality risk was 2.0 (95% CI, 1.1–3.8; *p* = 0.032) for patients with cancer cachexia. Beyond its well-recognized effect of deregulating the host’s metabolic homeostasis, cancer cachexia has also been associated with immune system dysfunction and increased susceptibility to infections [23]. Cachexia significantly impacts the efficacy of both chemotherapy and RT by leading to reduced survival rates [24]. The efficacy and safety of anamorelin, which is an orally active, high-affinity, selective agonist of the ghrelin receptor, have been demonstrated in cancer patients with cachexia [25,26]. Development of new drugs targeting inflammation and metabolic abnormalities caused by cancer cachexia is also anticipated. References to potential therapeutic interventions, including anamorelin and novel anti-cachexia agents, are presented here as hypothesis-generating observations to motivate future prospective intervention studies in this population, rather than as direct clinical recommendations. Our observational data cannot establish causality, and any clinical application of these findings must await prospective validation in controlled studies with standardized nutritional assessment and intervention protocols.

Treatment discontinuation occurs more frequently in underweight patients, and non-hematologic grade 3 or higher acute toxicity tends to be observed [27]. In this study, there was no statistically significant evidence of reduced treatment tolerance or increased late AEs in the underweight or cancer cachexia groups. However, the study might be underpowered for the detection of a modest effect. This finding should be interpreted with caution: the study was not powered to detect differences in AEs, the retrospective design limited systematic AE documentation, and the absolute differences observed (e.g., lower chemotherapy use in the cachexia group) suggest clinically meaningful differences in treatment intensity that may not have reached statistical significance due to limited sample size. Furthermore, factors other than the patient characteristics we evaluated may also influence the differences in outcomes between the non-underweight and underweight groups. The positive association between high BMI and survival outcomes is known as the “obesity paradox” [28]. This may be due to overweight or obesity potentially functioning as a nutritional reservoir or being associated with a lower frequency of sarcopenia [29,30,31]. The positive association between overweight and survival in HNC is consistent with evidence provided by previous single-center studies [32,33,34,35,36]. Underweight patients with HNC exhibit significantly lower OS, while overweight and obese patients tend to show favorable HNC-specific survival [37]. Since we did not assess overweight status in this study, the impact of overweight remains unclear; however, the association between underweight and worse outcomes is consistent with our findings. Nutritional deficits have a significant impact on mortality, morbidity, and quality of life [38]. We believe our findings are likely applicable to locally advanced HNC beyond HPC. Additionally, our findings highlight the importance of initiating proactive nutritional interventions prior to treatment initiation for locally advanced HNC patients with low BMI and cancer cachexia.

Most studies investigating the relationship between underweight or sarcopenia and prognosis in cancer patients evaluate only DFS or OS [2,3,4,5,9,10]. We believe our research holds value in that it assesses the impact of these three potential risk factors on LRC, which is essential for posttreatment quality of life in HNC patients. This study is also valuable in that it clearly demonstrates that underweight is associated with a higher risk of LRC. The absence of statistically significant prognostic association for sarcopenia in our study should not be interpreted as evidence that sarcopenia is clinically unimportant. Multiple methodological factors may limit our ability to detect such an association: the use of a single SMI threshold without adjustment for muscle quality or systemic inflammation, the relatively small subgroup size, and the high degree of overlap between sarcopenia and underweight in our cohort (55% of underweight patients also had sarcopenia). Regarding the SMI cutoff value used as the standard for sarcopenia, we adopted the same standard for East Asians, but this is also not absolute. Results may vary depending on the cutoff value, so further investigation is necessary going forward. We acknowledge that the lack of prognostic significance of sarcopenia alone in our cohort may be partly explained by the relatively crude single-parameter assessment using SMI and that composite indices integrating inflammatory markers, serum albumin, and muscle quality (myosteatosis) may provide greater prognostic resolution. This is now framed as an important direction for future research. The albumin–myosteatosis gauge is a novel integrated measure proposed to assess myosteatosis along with serum albumin level as a surrogate of systemic inflammation and malnutrition [39]. Future studies incorporating composite indices such as the albumin–myosteatosis gauge may better capture the prognostic signal of muscle-related nutritional deficiency.

The present study had several limitations. First, this is a single-center retrospective study; treatment modalities including RT technique and chemotherapy regimens were not standardized, and selection bias and residual confounding may exist despite multivariate adjustment. The long inclusion period (2004–2025) introduced temporal heterogeneity in terms of treatment approach. Unmeasured evolution in supportive care, nutritional management, and staging work-up over this period may have influenced results. In fact, in the multivariate analysis, the timing of treatment had a significant effect only on OS (Supplementary Table S2). Second, pretreatment weight-loss data were missing for 30% of patients, precluding cachexia assessment in this group. Although patients with missing data did not significantly differ from evaluable patients in key clinical characteristics except N-classification (Supplementary Table S1), residual selection bias cannot be fully excluded. Third, this study did not evaluate weight loss during RT, which is also an important prognostic indicator in HNC patients [40,41]. The retrospective cohort study by Langius et al. investigated the prognostic impact of weight loss, both before and during RT, on 5-year disease-specific survival (DSS) in 1340 HNC patients [40]. Critical weight loss during RT (defined as >5% by week 8 or >7.5% by week 12) was observed in 57% of patients and independently predicted worse DSS. The prospective observational study of Capuano et al. enrolled 40 locally advanced HNC patients undergoing concomitant chemoradiotherapy to examine how weight loss affects clinical outcomes. A body weight reduction exceeding 20% was significantly associated with treatment interruption, infections, early mortality, and hospital readmission [41]. As the exclusive focus on pretreatment assessments limits our ability to capture the full clinical picture, we frame the evaluation of longitudinal nutritional changes as a key objective for future prospective studies. Fourth, no formal power analysis was performed, and the non-significant findings for sarcopenia and AE analyses may partly reflect insufficient statistical power. Fifth, the East Asian SMI cutoff values used to define sarcopenia may not be optimal for all patients in our cohort and limit generalizability to non-Asian populations. Sixth, chemotherapy regimen heterogeneity was captured only as a binary variable in multivariate models, which may not fully account for differences in treatment intensity. While the inclusion of chemotherapy as a binary covariate captures whether systemic therapy was administered, it does not account for differences in regimen type (cisplatin, cetuximab, docetaxel, or induction protocols), dosing intensity, or number of cycles. This simplification may obscure differential treatment effects across subgroups, particularly as the cachexia group had a significantly lower rate of chemotherapy use (31% vs. 10%, *p* = 0.006).

## 5. Conclusions

We examined the association of pretreatment underweight, sarcopenia, and cancer cachexia with survival outcomes in HPC patients undergoing RT. The results show that pretreatment underweight and cancer cachexia are independent negative prognostic factors for LRC outcomes, as well as for DFS and OS. These associations are observational in nature and do not establish causality. No statistically significant difference in treatment tolerance or late AEs was observed; however, these analyses were limited by the retrospective design and potential lack of statistical power. Prospective studies with standardized nutritional assessment protocols, pre-specified intervention arms, and sufficient sample sizes are essential to validate these findings and to establish the clinical benefit of pre-treatment nutritional optimization in this patient population.

## Figures and Tables

**Table 1:** Patient and treatment characteristics.

Characteristics	All Patients	Underweight	Non-Underweight	*p*-Value	Cachexia	Non-Cachexia	*p*-Value
	*n* = 167	*n* = 76	*n* = 91		*n* = 45	*n* = 72	
Age (years)	69 (46–90)	69 (50–90)	70 (46–89)	0.87	71 (52–89)	70 (46–90)	0.048
Sex				<0.001			0.75
Male	149 (89%)	60 (79%)	89 (98%)		40 (89%)	66 (92%)	
Female	18 (11%)	16 (21%)	2 (2%)		5 (11%)	6 (8%)	
PS				0.25			0.012
0	101 (60%)	41 (54%)	60 (66%)		17 (38%)	47 (65%)	
1	54 (32%)	28 (37%)	26 (29%)		23 (51%)	21 (29%)	
2	12 (7%)	7 (9%)	5 (5%)		5 (11%)	4 (6%)	
Double cancer				0.83			0.34
No	140 (84%)	63 (83%)	77 (85%)		39 (87%)	57 (79%)	
Yes	27 (16%)	13 (17%)	14 (15%)		6 (13%)	15 (21%)	
Smoking *				0.79			0.53
Yes	146 (88%)	65 (85%)	81 (89%)		42 (93%)	63 (88%)	
No	17 (10%)	8 (11%)	9 (10%)		3 (7%)	8 (11%)	
BMI (kg/m^2^)	20.3 (13.8–46.2)	18.3 (13.8–19.9)	22.6 (20.0–46.2)	<0.001	18.7 (13.8–27.1)	21.0 (14.5–30.8)	<0.001
SMI (cm^2^/m^2^)	41.8 (24.2–66.3)	36.3 (24.2–52.9)	46.6 (30.0–66.3)	<0.001	38.0 (24.2–59.1)	43.8 (27.6–65.9)	<0.001
Sarcopenia				<0.001			0.010
Yes	54 (32%)	42 (55%)	12 (13%)		22 (49%)	18 (25%)	
No	113 (68%)	34 (45%)	79 (87%)		23 (51%)	54 (75%)	
BW change (%) **	−2.0 (−24.0–16.1)	−2.5 (−24.0–4.0)	−1.7 (−20.3–16.1)	0.090	−5.1 (−24.0–−2.0)	−0.3 (−4.8–16.1)	<0.001
Cachexia **				<0.001			NA
Yes	45 (27%)	34 (45%)	11 (12%)		45 (100%)	0	
No	72 (43%)	23 (30%)	49 (54%)		0	72 (100%)	
T-classification				0.54			0.77
1	22 (13%)	11 (14%)	11 (12%)		7 (16%)	9 (13%)	
2	50 (30%)	21 (28%)	29 (32%)		11 (24%)	22 (31%)	
3	37 (22%)	14 (18%)	23 (25%)		9 (20%)	16 (22%)	
4a,4b	58 (35%)	30 (39%)	28 (31%)		18 (40%)	25 (35%)	
N-classification				0.86			0.13
0	46 (28%)	23 (30%)	23 (25%)		9 (20%)	16 (22%)	
1	19 (11%)	5 (7%)	14 (15%)		1 (2%)	10 (14%)	
2	75 (45%)	34 (45%)	41 (45%)		23 (51%)	34 (47%)	
3	27 (16%)	14 (18%)	13 (14%)		12 (27%)	12 (17%)	
Stage				0.84			0.28
I	12 (7%)	7 (9%)	5 (5%)		4 (9%)	3 (5%)	
II	18 (11%)	5 (7%)	13 (14%)		2 (4%)	8 (14%)	
III	23 (14%)	10 (13%)	13 (14%)		3 (7%)	10 (14%)	
IVa, IVb	114 (68%)	54 (71%)	60 (67%)		36 (80%)	51 (67%)	
Chemotherapy				0.087			0.006
Use	133 (80%)	56 (74%)	77 (85%)		31 (69%)	65 (90%)	
Non-use	34 (20%)	20 (26%)	14 (15%)		14 (31%)	7 (10%)	
Treatment era				0.60			0.089
before 2012	45 (27%)	22 (29%)	23 (25%)		16 (36%)	15 (21%)	
after 2012	122 (73%)	54 (71%)	68 (75%)		29 (64%)	57 (79%)	
Radiation dose	70.0 (30.0–70.0)	70.0 (30.0–70.0)	70.0 (64.2–70.0)	0.066	70.0 (30.0–70.0)	70.0 (60.0–70.0)	0.28
<66 Gy	17 (17%)	11 (14%)	6 (7%)	0.12	6 (13%)	7 (10%)	0.56
≥66 Gy	150 (90%)	65 (86%)	85 (93%)		39 (87%)	65 (90%)	

Data are shown as *n* (%) or medians (range). * Smoking history was missing for 4 patients. ** Cachexia analysis was restricted to the 117 patients for whom pretreatment weight-loss data were available. The remaining 50 patients (30%) lacked pretreatment weight-loss data and could not be classified for cachexia status. PS, performance status; BMI, body mass index; SMI, skeletal muscle index; BW, body weight.

**Table 2:** Univariate analyses for locoregional control (LRC), disease-free survival (DFS), and overall survival (OS).

Characteristics	3-Year LRC (%)			3-Year DFS (%)			3-Year OS (%)		
	Rate	95% CI	*p*-Value	Rate	95% CI	*p*-Value	Rate	95% CI	*p*-Value
Age (years)			0.025			0.028			0.33
≤70	66	53–76		57	45–67		76	65–84	
>70	51	37–64		38	6.3–26		63	48–74	
Sex			0.90			0.74			0.72
Male	59	49–67		48	39–57		70	61–77	
Female	71	4–87		54	25–76		78	42–93	
PS			<0.001			<0.001			<0.001
0	67	56–76		58	46–67		79	69–87	
1	47	30–62		36	22–49		57	41–71	
2	38	6.7–71		34	6.3–66		37	1.5–78	
Double cancer			0.90			0.52			0.042
No	60	50–69		50	41–59		74	65–82	
Yes	56	31–75		41	21–60		52	30–70	
Smoking			0.46			0.089			0.25
Yes	57	47–65		44	35–53		68	59–76	
No	73	43–89		75	46–90		88	59–97	
Underweight			0.002			0.008			0.014
Yes	47	34–59		39	27–51		58	44–70	
No	70	57–80		56	44–67		80	67–87	
Sarcopenia			0.069			0.076			0.18
Yes	48	32–62		43	28–56		62	46–75	
No	65	53–74		51	40–61		74	63–82	
Cachexia			<0.001			<0.001			0.003
Yes	32	18–47		23	12–37		51	33–66	
No	69	54–80		53	39–66		76	62–86	
T-classification			0.25			0.097			0.26
1, 2	58	44–73		54	40–66		73	59–82	
3	67	46–78		52	33.6–68		71	52–84	
4	57	41–73		39	25–53		68	51–80	
N-classification			0.024			0.002			0.050
0	71	53–83		66	49–78		81	64–91	
1	66	35–85		56	28–76		67	37–85	
2	52	39–64		41	29–53		67	53–77	
3	59	34–77		36	16–57		66	40–83	
Stage			0.072			0.005			0.059
I, II	60	36–77		57	35–74		79	56–91	
III	75	49–89		72	47–86		85	59–95	
IVa	56	43–67		43	32–54		66	53–75	
IVb	59	34–77		36	16–57		66	40–83	
Chemotherapy			0.067			0.008			0.003
Use	62	52–70		52	43–61		75	65–82	
Non-use	45	20–67		29	11–51		51	27–71	
Treatment era			0.19			0.86			0.20
before 2012	65	48–78		50	39–59		58	41–72	
after 2012	57	45–66		46	31–61		77	67–84	
Radiation dose			0.60			0.60			0.62
≥66 Gy	59	49–68		50	40–58		72	63–80	
<66 Gy	61	33–80		41	19–63		56	28–76	

CI, confidence interval; PS, performance status.

## Data Availability

The datasets generated and/or analyzed during the present study are not publicly available due to ethical reasons, but are available from the corresponding author upon reasonable request.

## Supplementary Materials

The following supporting information can be downloaded at: https://www.mdpi.com/article/10.3390/cancers18081244/s1. Table S1: Comparison of patient and treatment characteristics between patients with and without available cachexia data; Table S2: Results of each multivariate analysis when underweight, sarcopenia, and cachexia were entered into the model separately; Table S3: Results of multivariate analysis using multiple imputation to address missing data for cachexia; Table S4: Results of multivariate analysis using a sensitivity analysis to address missing data for cachexia.

## Abbreviations

The following abbreviations are used in this manuscript:

3DCRT	three-dimensional conformal radiotherapy
AEs	adverse events
BMI	body mass index
CI	confidence interval
CTCAE	common terminology criteria for adverse events
DFS	disease-free survival
EPCRC	European palliative care research collaborative
EQD2	equivalent dose at 2-Gy
HNC	head and neck cancer
HPC	hypopharyngeal cancer
IMRT	intensity-modulated radiotherapy
LRC	locoregional control
OS	overall survival
PF	cisplatin and fluorouracil
PS	performance status
PTV	planning target volume
RT	radiation therapy
SCC	squamous-cell carcinoma
SMI	skeletal muscle index
TPF	docetaxel, cisplatin, and fluorouracil
