# Supplementary material for: Association of Underweight, Sarcopenia, and Cancer Cachexia with Survival Outcomes in Hypopharyngeal Cancer Radiotherapy
Source: Cancers (Basel). 2026 Apr 14;18(8):1244. doi: 10.3390/cancers18081244 (PMC13115260; doi:10.3390/cancers18081244)
Supplement: Supplementary file 1 [file cancers-18-01244-s001.zip › cancers-4240004-supplementary.pdf]

Supplementary Table S1. Comparison of patient and treatment characteristics between patients with and without available cachexia data.

Characteristics	Classifiable n = 117	Unclassifiable n = 50	*p*-value
Age (years)	70 (46–90)	67 (53–88)	0.49
Sex			0.42
Male	106 (91%)	43 (86%)	
Female	11 (9%)	7 (14%)	
PS			0.059
0	64 (55%)	37 (74%)	
1	44 (38%)	10 (20%)	
2	9 (8%)	3 (6%)	
Double cancer			0.49
No	96 (82%)	44 (88%)	
Yes	21 (18%)	6 (12%)	
Smoking history*			0.58
Yes	105 (90%)	41 (82%)	
No	11 (9%)	6 (12%)	
BMI (kg/m^2)	20.1 (13.8-30.8)	21.1 (14.6-46.2)	0.12
SMI (cm^2/m^2)	41.8 (24.2-65.9)	44.4 (26.4-66.3)	0.40
Sarcopenia			0.48
Yes	40 (34%)	14 (28%)	
No	77 (66%)	36 (72%)	
BW change (%)	-2.0 (-24.0-16.1)	NA	NA
Cancer cachexia			NA
Yes	45 (38%)	NA	
No	72 (62%)	NA	
T-classification			0.79
1	16 (14%)	6 (12%)	
2	33 (28%)	17 (34%)	
3	25 (21%)	12 (24%)	
4a,4b	43 (37%)	15 (30%)	

N-classification			0.005
0	25 (21%)	21 (42%)	
1	11 (9%)	8 (16%)	
2	57 (49%)	18 (36%)	
3	24 (21%)	3 (6%)	
Stage			0.071
I	7 (6%)	5 (10%)	
II	10 (9%)	8 (16%)	
III	13 (11%)	10 (20%)	
IVa, IVb	87 (74%)	27 (54%)	
Chemotherapy			0.29
Use	96 (82%)	37 (74%)	
Non-use	21 (18%)	13 (26%)	
Treatment era			0.85
Before 2012	31 (26%)	14 (28%)	
After 2012	86 (74%)	36 (72%)	
Radiation dose	70.0 (60.0-70.0)	70.0 (64.2-70.0)	0.40
< 66 Gy	13 (11%)	4 (8%)	0.78
≥ 66 Gy	104 (89%)	46 (92%)	

Data are shown as n (%) or medians (range). *Smoking history was missing for 4 patients.

PS, performance status; BMI, body mass index; SMI, skeletal muscle index; BW, body weight; NA, not applicable.

Supplementary Table S2. Results of each multivariate analysis when underweight, sarcopenia, and cachexia were entered into the model separately.

S2.1 Results after including underweight as a covariate.

	Locoregional control				Disease-free survival				Overall survival			
	HR	95% CI		*p*-value	HR	95% CI		*p*-value	HR	95% CI		*p*-value
Age (≦70 vs. >70)	1.8	1.03	3.1	0.037	1.7	1.1	2.8	0.020	1.5	0.87	2.7	0.14
Sex (Female vs. Male)	1.9	0.74	4.7	0.19	1.4	0.60	3.4	0.43	1.9	0.62	5.7	0.26
PS (0 vs. 1. vs. 2)	1.8	1.1	2.9	0.017	2.0	1.3	2.9	0.0005	1.8	1.1	3.0	0.012
Double cancer (No vs. Yes)	1.2	0.55	2.6	0.65	1.5	0.80	2.7	0.21	2.2	1.1	4.4	0.027
Smoking (No vs. Yes)	1.5	0.59	3.9	0.39	2.5	0.94	6.4	0.065	1.6	0.45	5.4	0.48
T-stage (1,2 vs. 3 vs. 4)	0.88	0.65	1.2	0.43	1.1	0.83	1.4	0.56	1.2	0.86	1.7	0.30
N-stage (0 vs. 1 vs. 2 vs. 3)	1.5	1.14	2.0	0.004	1.5	1.2	2.0	0.0004	1.4	1.1	1.9	0.019
Chemotherapy (Use vs. No)	1.0	0.48	2.1	1.0	1.3	0.67	2.4	0.47	1.7	0.81	3.7	0.16
Treatment era (2012- vs. -2012)	0.85	0.40	1.8	0.66	1.2	0.67	2.1	0.54	2.3	1.2	4.5	0.013
Radiation dose (≥ 66 vs. < 66)	0.83	0.29	2.4	0.73	1.1	0.48	2.3	0.90	0.8	0.32	1.9	0.56
Underweight (No vs. Yes)	2.6	1.5	4.5	<0.001	1.9	1.2	3.0	0.007	1.9	1.1	3.4	0.030

HR, hazard ratio; CI, confidence interval; PS, performance status.

S2.2 Results after including sarcopenia as a covariate.

	Locoregional control				Disease-free survival				Overall survival			
	HR	95% CI		*p*-value	HR	95% CI		*p*-value	HR	95% CI		*p*-value
Age (≦70 vs. >70)	1.7	0.97	2.8	0.065	1.6	1.0	2.6	0.036	1.3	0.78	2.3	0.30
Sex (Female vs. Male)	1.0	0.40	2.3	0.92	1.0	0.43	2.2	0.96	1.3	0.45	3.8	0.62
PS (0 vs. 1. vs. 2)	1.8	1.1	2.9	0.016	1.9	1.3	2.8	0.0006	1.9	1.2	3.1	0.006
Double cancer (No vs. Yes)	1.1	0.53	2.4	0.75	1.4	0.77	2.6	0.27	2.1	1.1	4.2	0.034
Smoking (No vs. Yes)	1.6	0.63	4.2	0.31	2.5	0.97	6.5	0.059	1.7	0.49	5.9	0.40
T-stage (1,2 vs. 3 vs. 4)	0.93	0.68	1.3	0.65	1.1	0.86	1.5	0.41	1.2	0.90	1.7	0.19
N-stage (0 vs. 1 vs. 2 vs. 3)	1.4	1.10	1.9	0.008	1.5	1.2	1.9	0.0007	1.4	1.01	1.8	0.041
Chemotherapy (Use vs. No)	1.1	0.51	2.2	0.89	1.4	0.72	2.5	0.34	2.0	0.94	4.1	0.071
Treatment era (2012- vs. -2012)	0.73	0.35	1.5	0.40	1.1	0.62	2.0	0.74	2.2	1.1	4.3	0.018
Radiation dose (≥ 66 vs. < 66)	1.2	0.42	3.2	0.78	1.2	0.58	2.6	0.59	0.95	0.41	2.2	0.91
Sarcopenia (No vs. Yes)	1.6	0.96	2.8	0.072	1.3	0.85	2.1	0.21	1.1	0.63	2.0	0.72

HR, hazard ratio; CI, confidence interval; PS, performance status.

S2.3 Results after including cachexia as a covariate.

	Locoregional control				Disease-free survival				Overall survival			
	HR	95% CI		*p*-value	HR	95% CI		*p*-value	HR	95% CI		*p*-value
Age (≦70 vs. >70)	1.2	0.65	2.2	0.56	1.3	0.76	2.2	0.35	1.1	0.59	2.0	0.77
Sex (Female vs. Male)	1.1	0.40	3.1	0.83	1.2	0.44	3.2	0.73	1.7	0.45	6.7	0.43
PS (0 vs. 1. vs. 2)	1.9	1.1	3.3	0.028	1.8	1.1	2.9	0.012	1.7	0.94	3.0	0.083
Double cancer (No vs. Yes)	1.7	0.71	4.2	0.22	1.5	0.75	3.2	0.24	2.4	1.03	5.5	0.043
Smoking (No vs. Yes)	0.98	0.37	2.6	0.97	1.7	0.63	4.5	0.30	1.1	0.30	4.1	0.88
T-stage (1,2 vs. 3 vs. 4)	1.2	0.84	1.8	0.30	1.5	1.1	2.1	0.010	1.6	1.1	2.4	0.015
N-stage (0 vs. 1 vs. 2 vs. 3)	1.5	1.1	2.1	0.017	1.6	1.2	2.2	0.003	1.5	1.01	2.1	0.045
Chemotherapy (Use vs. No)	0.76	0.31	1.9	0.54	1.3	0.59	3.0	0.50	2.2	0.85	5.4	0.10
Treatment era (2012- vs. -2012)	0.64	0.28	1.5	0.29	0.92	0.48	1.7	0.79	2.1	0.97	4.5	0.061
Radiation dose (≥ 66 vs. < 66)	1.1	0.35	3.4	0.87	1.5	0.66	3.6	0.32	1.3	0.49	3.3	0.62
Cachexia (No vs. Yes)	3.4	1.8	6.3	<0.001	2.4	1.4	4.1	<0.001	2.0	1.1	3.8	0.032

HR, hazard ratio; CI, confidence interval; PS, performance status.

Supplementary Table S3. Results of multivariate analysis using multiple imputation to address missing data for cachexia.

	Locoregional control				Disease-free survival				Overall survival			
	HR	95% CI		*p*-value	HR	95% CI		*p*-value	HR	95% CI		*p*-value
Age (≦70 vs. >70)	1.6	0.88	2.7	0.12	1.6	0.96	2.5	0.07	1.2	0.70	2.2	0.45
Sex (Female vs. Male)	0.91	0.36	2.3	0.84	1.1	0.47	2.7	0.78	1.5	0.48	4.5	0.49
PS (0 vs. 1. vs. 2)	1.5	0.90	2.5	0.12	1.7	1.1	2.6	0.011	1.8	1.1	3.0	0.019
Double cancer (No vs. Yes)	1.6	0.72	3.5	0.24	1.7	0.92	3.2	0.088	2.7	1.3	5.6	0.008
Smoking (No vs. Yes)	1.6	0.58	4.4	0.35	2.3	0.85	6.1	0.10	1.6	0.44	5.9	0.46
T-stage (1,2 vs. 3 vs. 4)	1.04	0.75	1.5	0.80	1.2	0.89	1.6	0.24	1.3	0.92	1.9	0.13
N-stage (0 vs. 1 vs. 2 vs. 3)	1.5	1.1	2.0	0.009	1.6	1.2	2.1	<0.001	1.4	1.1	2.0	0.022
Chemotherapy (Use vs. No)	0.69	0.31	1.5	0.35	1.1	0.56	2.2	0.76	1.8	0.82	3.9	0.14
Treatment era (2012- vs. -2012)	0.55	0.25	1.2	0.13	0.92	0.51	1.7	0.79	2.0	1.01	4.1	0.047
Radiation dose (≥ 66 vs. < 66)	1.2	0.42	3.4	0.73	1.3	0.61	3.0	0.45	1.0	0.42	2.4	0.98
Cachexia (No vs. Yes)	4.1	2.1	8.0	<0.001	2.8	1.6	4.9	<0.001	2.2	1.1	4.4	0.026

HR, hazard ratio; CI, confidence interval; PS, performance status.

Supplementary Table S4. Results of multivariate analysis using a sensitivity analysis to address missing data for cachexia.

S4.1 Results when missing cachexia data are all classified as non-cachexia.

	Locoregional control				Disease-free survival				Overall survival			
	HR	95% CI		*p*-value	HR	95% CI		*p*-value	HR	95% CI		*p*-value
Age (≦70 vs. >70)	1.4	0.82	2.4	0.22	1.4	0.88	2.3	0.15	1.2	0.68	2.1	0.55
Sex (Female vs. Male)	1.02	0.42	2.5	0.97	1.2	0.51	2.8	0.68	1.4	0.48	4.2	0.53
PS (0 vs. 1. vs. 2)	1.6	0.96	2.5	0.073	1.7	1.2	2.5	0.006	1.8	1.1	2.9	0.018
Double cancer (No vs. Yes)	1.2	0.59	2.7	0.57	1.4	0.78	2.6	0.25	2.3	1.1	4.6	0.020
Smoking (No vs. Yes)	1.6	0.63	4.2	0.31	2.3	0.90	6.0	0.081	1.7	0.48	5.8	0.42
T-stage (1,2 vs. 3 vs. 4)	1.1	0.77	1.5	0.74	1.2	0.93	1.6	0.16	1.3	0.93	1.8	0.12
N-stage (0 vs. 1 vs. 2 vs. 3)	1.4	1.04	1.8	0.027	1.5	1.2	1.9	0.002	1.3	1.00	1.8	0.047
Chemotherapy (Use vs. No)	0.95	0.45	2.0	0.88	1.4	0.74	2.7	0.31	2.0	0.96	4.3	0.063
Treatment era (2012- vs. -2012)	0.60	0.29	1.3	0.19	0.97	0.55	1.7	0.93	2.1	1.1	4.1	0.033
Radiation dose (≥ 66 vs. < 66)	1.2	0.45	3.3	0.70	1.4	0.66	2.9	0.38	1.01	0.43	2.3	0.99
Cachexia (No vs. Yes)	3.1	1.8	5.3	<0.001	2.4	1.5	3.9	<0.001	1.9	1.1	3.3	0.022

HR, hazard ratio; CI, confidence interval; PS, performance status.

S4.2 Results when missing cachexia data are all classified as cachexia.

	Locoregional control				Disease-free survival				Overall survival			
	HR	95% CI		*p*-value	HR	95% CI		*p*-value	HR	95% CI		*p*-value
Age (≦70 vs. >70)	1.6	0.95	2.8	0.075	1.6	1.02	2.6	0.041	1.3	0.76	2.3	0.33
Sex (Female vs. Male)	0.98	0.40	2.4	0.97	1.05	0.46	2.4	0.92	1.4	0.47	4.0	0.56
PS (0 vs. 1. vs. 2)	1.8	1.1	2.8	0.015	1.9	1.3	2.8	0.001	1.9	1.2	3.0	0.005
Double cancer (No vs. Yes)	1.4	0.66	2.9	0.39	1.5	0.84	2.8	0.16	2.4	1.2	4.7	0.016
Smoking (No vs. Yes)	1.9	0.75	5.0	0.17	2.7	1.04	7.0	0.041	1.8	0.52	6.2	0.36
T-stage (1,2 vs. 3 vs. 4)	0.94	0.69	1.3	0.70	1.1	0.85	1.4	0.44	1.2	0.88	1.7	0.22
N-stage (0 vs. 1 vs. 2 vs. 3)	1.5	1.2	2.0	0.002	1.6	1.2	2.0	0.000	1.4	1.05	1.9	0.024
Chemotherapy (Use vs. No)	0.92	0.44	1.9	0.82	1.3	0.68	2.4	0.45	1.9	0.92	4.0	0.083
Treatment era (2012- vs. -2012)	0.74	0.35	1.5	0.42	1.1	0.64	2.0	0.69	2.2	1.2	4.4	0.017
Radiation dose (≥ 66 vs. < 66)	1.2	0.42	3.1	0.78	1.3	0.60	2.7	0.55	0.95	0.41	2.2	0.90
Cachexia (No vs. Yes)	2.0	1.1	3.5	0.017	1.5	0.93	2.3	0.10	1.4	0.82	2.5	0.21

HR, hazard ratio; CI, confidence interval; PS, performance status.
